# Time trends of cancer incidence in childhood in Campania region: 25 years of observation

**DOI:** 10.1186/s13052-016-0287-y

**Published:** 2016-09-06

**Authors:** Paolo Indolfi, Serena Picazio, Silverio Perrotta, Francesca Rossi, Andrea Pession, Martina Di Martino, Elvira Pota, Daniela Di Pinto, Cristiana Indolfi, Roberto Rondelli, Francesco Vetrano, Fiorina Casale

**Affiliations:** 1 Servizio di Oncologia Pediatrica, Dipartimento della Donna, del Bambino e di Chirurgia Generale e Specialistica- Seconda Università degli Studi di Napoli, Naples, Italy; 2 AIL Sezione “Valentina Picazio”, Caserta, Italy; 3 Unità di Oncologia ed Ematologia Pediatrica “Lalla Seràgnoli”, Università di Bologna, Bologna, Italy; 4 Responsabile Registro Tumori Infantili Regione Campania, Naples, Italy

**Keywords:** Epidemiology, Cancer, Children

## Abstract

**Background:**

Childhood cancer is relatively uncommon and the European age-standardized rate was 164 new case per million per year among children 0 to 14 years of age (95 % CI 158-170). Aims of our study are to evaluate the cases of these malignant diseases observed between 0 and 15 years of age in the Campania region between 1990 and 2014, the ration between observed and expected cases by disease and province of residence. Also we studied the percentage of extra-regional migration over the time by disease and province of residence.

**Methods:**

In this study we reported the patients with malignant disease observed in 25 years (1990–2014) based on the specialized registry, the Mod. 1.01 of the AIEOP (Association Italian Pediatric Hematology-Oncology). The size of the monitored population also allowed us to systematically examine five time trends: 1990–94: 1995–99; 2000–04; 2005–09; and 2010–14.

**Results:**

Between 1990 and 2014 a total of 3655 malignant neoplasms were reported: Napoli province (2059 cases), Salerno province (625), Caserta province (589), Avellino province (229), and Benevento province (153). Epidemiological data suggested that about 4100 cases could be expected in Campania region during the same period. The overall ratio between observed (O) and expected (E) numbers of cases in the five periods considered rose gradually from 0.69 in the first period to 0.76, then 0.82, 0.91, and 0.94, in the other periods considered. The extra-regional migration involved 1029 cases (28.1 %), showing a reduction from 33.7 % of the first period to 20.3 % of the last period considered. Considering single province of residence we observed the lowest rate of migration in Napoli and Caserta province, whereas higher levels were observed in the other provinces. For all provinces, except Salerno, the extra-regional migration declined significantly over time.

**Conclusions:**

The present findings showed an increase over time of O/E ratio, probably due to improvement in the organization of centers and greater trust of families in local centers. It is possible to further improve the efficiency of healthcare system of Campania region and migration can be reduced with a more rational use of hospitals throughout region.

## Background

Childhood cancer is relatively uncommon and actually the European age-standardized rate was about 164 new case per million per year among children 0 to 14 years of age (95 % CI 158-170) [1, 2]. Between 1988 and 2008 a total of 5,709 malignant neoplasms were reported in children aged 0–14 years. Incidence rates in children for all malignant cancer peaked in 1997 (211 cases/million boys and 175 cases/million girls per year), followed by a decline, non statistically significant [1, 2]. Instead, the annual incidence rate in adolescents (15–19 years) was 269 per million (95 % CI 256-283) [3, 4]. The ranking of cancer types as defined by the ICCC-3 (*International Classification for Childhood Cancer*) classification shows that leukemias are the most frequent neoplasms (33 % of all malignant cancers), followed by lymphomas (16 %), malignant tumours of Central Nervous System (CNS) (13 %), which increase to 18 % when including non-malignant tumours), followed by neoplasms of the peripheral nervous system (8 %), and the soft tissue cancers (7 %). The remaining neoplasms, more rare, account for no more than 5 % each and 23 % altogether [5, 6]. Aims of our study are to evaluate the incidence of these malignant diseases observed between 0 and 15 years of age in the Campania region, the ration between observed and expected cases by disease and province of residence. Also we studied the percentage of extra-regional migration over the time by disease and province of residence. A minor objective was to evaluate an eventually increase of incidence of cancer in Napoli and Caserta provinces, called “Ground of fires”, from involuntuary exposure to environmental pollutants. However, it is worth remembering that a correlation between pollution and cancer requires several types of evidence and a systematic approach following well-defined criteria and methods that make it a specialized activity.

## Methods

For this report we selected all cancer cases diagnosed in the 0–15 years age group. The age specific rates were computed dividing the number of newly diagnosed cases in a period in this group of age of population by the corresponding population in the same period. The rates defines the mean number of diagnoses in one year and is referred to 1,000,000 subjects of the specific age group. The European age-standardized rate was 164 new cases per million children per year (95 % CI 158-170) [5] In this study we reported the number of patients with malignant disease diagnosed before their 15th birthday and observed in 25 years (1990–2014), based on the specialized registry, the Mod. 1.01 of the AIEOP (*Association Italian Pediatric Hematology-Oncology*). This centralized archive, called Model (Mod.) 1.01, has been in use since 01.01.1989 and records essential standardized information on all cases of childhood cancer for ages 0–19 years, diagnosed and/or treated in the AIEOP centers. For this reason we started our analysis since 1990. We recognize the limits of the Mod. 1.01 as a potential cancer registry. An study was estimated for the period 1989–1998, as a total and by tumour type, comparing the recruitment *vs* the expected numbers based on the incidence rates measured by the Childhood Cancer Registry of Piedmont. Results showed good correspondence [7]. Actually the AIEOP database to include in Italy about the 90 % of children aged 0–15 years affected by cancer. The size of the monitored population also allowed us to systematically examine five time trends: 1990–94; 1995–99; 2000–04; 2005–09; and 2010–14. The evaluation of expected cases is considered related to the population in a province and the number of children residents in the same province. In detail, the global number of people in Campania region is of 5,790,929 and of 1,014,796 between 0–15 years old. Corresponding global population and 0–15 years people, were respectively for Napoli 3,086,622 and 572,335; Salerno 1,090,934 and 173,335; Caserta 886,758 and 160,277; Avellino 437,414 and 65,566; and Benevento 289,201 and 43,355 residents. (Specialized Registry for childhood Campania Region). Based on these numbers of children the expected cases of malignant neoplasm per year were for province of Napoli (93 cases); Salerno (28 cases); Caserta (26 cases); Avellino (10 cases); and Benevento (7 cases). The ranking of cancer types as defined by ICCC-3 show per year solid cancer non CNS as the most frequent neoplasms (61 cases), followed by leukemias (52 cases), CNS tumours (41 cases), and Hodgkin/non Hodgkin lymphomas (23 cases) [5]. We have calculated the number of observed cases (O) in the long time in Campania region and then we have analyzed the ratio between observed and expected (E) numbers of cases. The Breslow-ay test was used to assess O/E trends by diagnostic period. The aim was to check an eventually better O/E ratio over time to demonstrate that more cases have been treated at local centers. A secondary aim was to verify an eventually surplus of malignant cancer cases observed in the specific 5-year time and/or in the several provinces of Campania region. Finally, we have evaluated the extra-region migration as the number of the children affected by malignant neoplasm diagnosed and/or treated in other regions (or Countries) from where they live.

## Results

### Global analysis

During the study period 3655 cancer patients aged 0–15 years old were registered in the AIEOP database, 2036 males (55.7 %) and 1619 females (44.2 %). Epidemiological data suggest that about 4.100 cases in Campania Region and 37,000 cases in Italy could be expected during the same period. Table 1 shows all cases registered by diagnosis and province of residence in the five diagnostic period considered. Table 2 shows in detail all cases observed by type of disease in the five diagnostic period considered.

**Table 1:** Characteristics and numbers of children (0–15 years old) diagnosed with cancer in Campania Region between 1990 and 2014, registered in the AIEOP Mod. 1.01

Diagnostic period	No (%) of patients
1990–1994	611 (16.7)
1995–1999	673 (18.4)
2000–2004	726 (19.8)
2005–2009	806 (22.0)
2010–2014	839 (22.9)
Sex	
Male	2036 (55.7)
Female	1619 (44.2)
Type of Disease	
Acute lymphoblastic/hybrid Leukemia	1036 (28.3)
Acute non lymphoblastic Leukemia	220 (6.0)
Chronic Myeloid Leukemia	24 (0.6)
Non Hodgkin Lymphoma	281 (7.6)
Hodgkin Lymphoma	189 (5.1)
CNS Tumours	500 (13.6)
No CNS Tumours	1405 (38.4)
Province of residence	
Napoli	2059 (56.3)
Salerno	625 (17.0)
Caserta	589 (16.1)
Avellino	229 (6.2)
Benevento	153 (4.1)

**Table 2:** Observed malignant cancer cases by disease and period of observation (%)

Disease	1990–1994	1995–1999	2000–2004	2005–2009	2010–2014
ALL	210 (20.5)	193 (18.8)	205 (20.0)	199 (19.4)	217 (21.1)
LA hybrid/undiff.	6 (50.0)	4 (33.3)	1 (8.3)	1 (8.3)	0
ANLL	39 (17.7)	42 (19.0)	40 (18.1)	50 (22.7)	49 (22.2)
MCL	5 (20.8)	3 (12.5)	3 (12.5)	4 (16.6)	9 (37.5)
NHL	52 (18.5)	63 (22.4)	59 (20.9)	61 (21.7)	46 (16.3)
HL	16 (8.4)	35 (18.5)	35 (18.5)	41 (21.6)	62 (32.8)
Neuroblastoma	75 (21.9)	58 (16.9)	67 (19.5)	72 (21)	70 (20.4)
CNS Tumour	31 (6.2)	78 (15.6)	118 (23.6)	122 (24.4)	151 (30.5)
Kidney Tumour	44 (21.7)	45 (22.2)	29 (14.3)	39 (19.3)	45 (22.2)
Soft tissue sarcoma	31 (14.6)	44 (20.7)	45 (21.2)	56 (26.4)	36 (16.9)
Histiocytosis	25 (14.8)	27 (16.0)	33 (19.6)	47 (27.9)	36 (21.4)
GCT	15 (14.0)	18 (16.8)	12 (11.2)	29 (27.1)	33 (30.8)
S. Ewing/PNET	14 (12.8)	20 (18.3)	22 (20.1)	22 (20.1)	31 (28.4)
Retinoblastoma	16 (20.7)	9 (11.6)	22 (28.5)	20 (25.9)	10 (12.9)
Bone Tumour	16 (25.3)	12 (19.0)	8 (12.6)	14 (22.2)	13 (20.6)
Liver Tumour	6 (13.3)	12 (26.6)	9 (20.0)	8 (17.7)	10 (22.2)
Nasofaryngeal carcinoma	3 (33.3)	1 (11.1)	2 (22.2)	2 (22.2)	1 (11.1)
Adrenocortical carcinoma	3 (27.2)	2 (18.1)	3 (27.2)	0	3 (27.2)
Other Tumours	4 (6.6)	7 (11.6)	13 (21.6)	19 (31.6)	17 (28.3)
Total	611 (16.7)	673 (18.4)	726 (19.8)	806 (22)	839 (22.9)

*ALL* acute lymphoblastic leukemia, *ANLL* acute non lymphoblastic leukemia, *MCL* myeloid chronic leukemia, *NHL* non hodgkin lymphoma, *HL* hodgkin lymphoma, *CNS* central nervous system, *GCT* germ cell tumour, *PNET* peripheral neuroectodermal tumor

### O/E ratio cases by type of cancer and period of observation

Related to the European age-standardized rate of about 164 new case per million per year among children 0–15 years and the ranking of cancer type as defined by the ICCC-3 we expected in Campania Region every five years time period about 260 cases of Leukemias; 115 cases of Lymphomas; 305 cases of Solid tumours (No CNS); and 205 cases of CNS tumours. Table 3 shows all cases observed and the O/E ratios in the five diagnostic period considered. The O/E ratio rose gradually from the first 5-year time to the last period for Lymphomas, CNS tumours, and Solid tumours (No CNS); the improvement was statistically significant for CNS tumours. The O/E ratio was particularly high for Leukemias.

**Table 3:** Ratio between observed and expected cases by type of cancer and period of observation (%)

Disease		1990–1994		1995–1999	2000–2004	2005–2009	2010–2014
	E	O	O/E	O/E	O/E	O/E	O/E
Leukemias	260	260	1.0	0.93	0.95	0.97	1.0
Limphomas	115	68	0.59	0.85	0.81	0.88	0.93
CNS tumours	205	31	0.15	0.38	0.57	0.59	0.73*
Solid tumours (no CNS)	305	252	0.82	0.83	0.86	1.0	1.0

*CNS* central nervous system, *E* expected cases/five years, *O* observed cases/five years

*p < 0.05 for trend across diagnostic periods

### O/E ratio cases by province of residence at diagnosis and period of observation

Based on the number of children living in Campania region the expected cases of malignant neoplasm every five years of observation were 465 for province of Napoli, 140 for Salerno, 130 for Caserta, 50 for Avellino, and 35 for Benevento. Table 4 shows the O/E ratios in the five diagnostic periods considered. The overall O/E ratio rose gradually from the first period to the last period considered, with the exception of Benevento province. This improvement was statistically significant for Napoli and Caserta provinces, as trend across diagnostic periods. In the Caserta and Salerno provinces the O/E ratio was particularly high (>1) in the last 5-year time.

**Table 4:** Ration between observed and expected cases by province of residence at diagnosis and period of observation (%)

Province of **r**esidence		1990–1994		1995–1999	2000–2004	2005–2009	2010–2014
	E/5 years	O	O/E	O/E	O/E	O/E	O/E
Napoli	465	344	0.73	0.82	0.90	0.98	0.96*
Caserta	130	101	0.77	0.71	0.83	0.99	1.2*
Salerno	140	97	0.69	0.84	0.85	0.94	1.1
Avellino	50	40	0.80	1.0	0.80	1.0	0.90
Benevento	35	29	0.82	0.74	1.0	0.94	0.77

*E* expected cases/five years, *O* observed cases/five years

*p < 0.05 for trend across diagnostic periods

### Extra-regional migration by period of observation and type of disease

Analyzing the 3655 cases who were diagnosed in the period 1990–2014, extra-regional migration involved 1029 (28.1 %) cases. Table 5 shows the global extra-regional migration for all cases, by disease. Table 6 shows in detail the variation in the five diagnostic periods considered of all malignat neoplasms observed. The analysis over time proves a progressive improvement for more frequent pediatric neoplasms. In detail, we observed for acute/hybrid lymphoblastic leukemias a progressive reduction of extra-regional migration from 18.5 % of the first period examined to 10.1 % of the last 5-year time (2010–2014), as for acute non lymphoblastic leukemia from 38.4 % to 12.2 %, Also, we observed a very good reduction of migration over time for lymphomas, neuroblastoma and kidney cancer, with a reduction from 36.5 %, 42.6 %, and 31.8 % for a first 5-year time to 17.7 %, 12.8 %, and 13.3 %, respectively. A minor improvement over time was registered for a soft tissue sarcomas with a percentage of reduction from 38.7 % to 22.2 % of the 2010–2014 period. Actually, non-satisfactory results were related to CNS tumors and Ewing/PNET sarcomas with a percentage reduction from 80.6 % and 64.2 % to 35 % and 54.8 %, respectively. Different is the problem related to the retinoblastoma, probably due to the more specific diagnostic and therapeutic approach. In fact, we observed a progressive extra-regional migration over time from a 12.5 % of the period 1990–1994 to 100 % of the 2010–2014 (Table 6).

**Table 5:** Extra-regional migration of the 3655 patients studied by disease and province of residence

Disease	No patients/total (%)
Acute lymphoblastic/hybrid Leukemia	143/1036 (13.8)
Acute non lymphoblastic Leukemia	37/220 (16.8)
Chronic Myeloid Leukemia	3/24 (12.5)
Non Hodgkin Lymphoma	73/281 (25.9)
Hodgkin Lymphoma	47/189 (24.8)
Neuroblastoma	93/342 (28.9)
CNS Tumours	251/500 (50.2)
Kidney Tumours	44/202 (21.7)
Soft Tissue Sarcoma	70/212 (33.0)
Histiocytosis	40/168 (23.8)
Germinal Cell Tumours	24/107 (22.4)
S. Ewing/PNET	57/109 (52.2)
Retinoblastoma	50/77 (64.9)
Bone Tumours	42/63 (66.6)
Liver Tumours	22/45 (48.8)
Other Tumours	27/60 (45.0)
Total	1029/3655 (28.1)
Province of residence	
Napoli	392/2059 (19.0)
Salerno	266/625 (42.5)
Caserta	175/589 (29.7)
Avellino	104/229 (45.4)
Benevento	68/153 (44.4)

*CNS* central nervous system, *PNET* peripheral neuroectodermal tumor

**Table 6:** Extra-regional migration by period of observation and type of disease (%)

Disease	1990–1994	1995–1999	2000–2004	2005–2009	2010–2014
ALL	40 (18.5)	29 (15)	29 (14.7)	23 (11.1)	22 (10.1)
ANLL	15 (38.4)	7 (16.6)	3 (7.5)	7 (14)	6 (12.2)
MCL	1 (20.0)	1 (33.3)	1 (33.3)	0	0
NHL	19 (36.5)	22 (34.9)	18 (30.5)	12 (19.6)	2 (4.3)
HL	5 (31.2)	8 (22.8)	14 (40.0)	9 (21.9)	11 (17.7)
Neuroblastoma	32 (42.6)	22 (37.9)	17 (25.3)	19 (26.3)	9 (12.8)
CNS Tumour	25 (80.6)	40 (51.2)	67 (56.7)	66 (54)	53 (35.0)
Kidney Tumour	14 (31.8)	10 (22.2)	7 (24.1)	7 (17.9)	6 (13.3)
Soft tissue sarcoma	12 (38.7)	10 (22.7)	20 (44.4)	20 (35.7)	8 (22.2)
Histiocytosis	4 (16.0)	7 (25.9)	10 (30.3)	10 (21.2)	9 (25.0)
GCT	4 (26.6)	3 (16.6)	4 (33.3)	7 (24.1)	6 (18.1)
S. Ewing/PNET	9 (64.2)	14 (70.0)	8 (36.3)	9 (40.9)	17 (54.8)
Retinoblastoma	2 (12.5)	3 (33.3)	17 (77.2)	18 (90)	10 (100)
Bone Tumour	15 (93.7)	11 (91.6)	6 (75.0)	7 (50)	3 (23.0)
Liver Tumour	4 (66.6)	7 (58.3)	4 (44.4)	4 (50)	3 (30.0)
Other Tumours	5 (50.0)	6 (60.0)	4 (22,2)	5 (23.8)	7 (33.3)
Total	206/611 (33.7)	200/673 (29.7)	229/726 (31.5)	223/806 (27.6)	171/839 (20.3)

*ALL* acute lymphoblastic leukemia, *ANLL* acute non lymphoblastic leukemia, *MCL* myeloid chronic leukemia, *NHL* non hodgkin lymphoma, *HL* hodgkin lymphoma, *CNS* central nervous system, *GCT* germ cell tumour, *PNET* peripheral neuroectodermal tumor

### Extra-regional migration by province of residence

Table 5 shows the extra-regional migration of the patients by province of residence. In Table 7 was evident the reduction of migration over time in the five provinces of Campania region even if with different results. Better results were registered for Napoli and Caserta whereas in the last 5-year time considered in the other three provinces more cases opted for the diagnosis and/or treatment in centers of a different region: Salerno (36.7 %); Avellino (26.6 %), and Benevento (25.9 %).

**Table 7:** Extra-regional migration by province of residence at diagnosis and period of observation of the 1029 patients examined (%)

Province of residence	1990–1994	1995–1999	2000–2004	2005–2009	2010–2014
Napoli	85 (24.7)	72 (18.7)	92 (21.9)	82 (17.8)	61 (13.5)
Caserta	32 (31.6)	31 (33.3)	40 (37.0)	39 (30.2)	33 (20.8)
Salerno	44 (45.3)	53 (44.9)	56 (46.6)	55 (41.6)	58 (36.7)
Avellino	21 (52.5)	23 (45)	19 (47.5)	29 (54.7)	12 (26.6)
Benevento	17 (58.6)	12 (46.1)	18 (47.3)	14 (42.4)	7 (25.9)

## Discussion

The present findings update descriptive cancer epidemiology in children (0–15 years) in Campania in the period 1990–2014 (25 years of observation) based on data provided by specialized clinical Mod.1.01 from AIEOP centers. The cases of cancer expected in Campania in this period, calculated using the last AIRTUM’s site-specific incidence rates by age group (0–15 years), were about 4,100 cases (11.5 %), of which 29.7 % were leukemias, 14.5 % were lymphomas, 20.4 % were CNS tumours, and 35.4 % were solid neoplasms non CNS. These percentages bring about a number of expected cases per year of 51 cases of leukemia, 25 cases of lymphoma, 35 cases of CNS tumours, and 61 cases of solid cancer non CNS. The present study shows that O/E ratio for leukemias and solid neoplasms non CNS was high since 1990 to demonstrate the efficacy of AIEOP organization and Mod. 1.01 for these diseases. Instead, the improvement of observation for lymphomas was more gradual over time and a increase of O/E ratio was observed from 0.59 in the period 1990–1994 to the most cases have recently been treated at local centers AIEOP. Recently, better O/E ratio was evident for CNS tumours with a two thirds cured in local centers, respect to the 0.15–0.38 O/E ratio observed in the 1990–2000 period. These results probably are due to substantial improvements in non-invasive diagnostic techniques but also to better collaboration between pediatric and neuro-surgery oncologists. These data were calculated using the Italian Network of Cancer Registries (AIRTUM) that includes 32 general cancer registries and five specialized which two dedicated to childhood (0–14 years), including about 4,000,000 of children. According to the province of residence in Campania in our study the overall O/E ratio rose gradually from the first time to last 5-year time and in 2010–2014 this ratio was particularly high (>1) for cancer observed in province of Caserta and Salerno. Conversely, the lowest O/E ratios were seen in province of Benevento. The analysis of extra-regional migration shows a progressive improvement of reduction over time, particularly for emo-lymphoproliferative diseases, neuroblastoma, and kidney cancer (under 20 % in the last period). Actually, there is still a relevant elective migration (more of 30 %) for CNS tumours, Ewing/PNET sarcomas, liver and rare tumours. This situation is motivated by organizational shortcomings which have created a historically rooted distrust toward health centers in their home region. This is particularly true for retinoblastoma were the rarity of neoplasm and specific local diagnostic and therapeutic approach makes migration toward another region obligatory. Our data are according to AIRTUM registry where the extra-regional migration of Campania involved 31.1 % of cases in the five-year period 2001–2005 *vs* 35.5 % of our patients and 25.6 % of cases during the next five years (2006–2010) *vs* 27.6 % of our observation. Better results (20.3 %) were registered in our region in the last period considered [5, 6]. The evaluation of migration by province of residence describes a progressive reduction over time in the different provinces even if the better results were registered for Napoli and Caserta. Minor results have been registered for Benevento and Avellino, while the lowest rate of reduction was observed in Salerno with a decrease of 9 % at last period respect to the percentage of the first period 1990–1994. In a 2005 Censis study on the reason behind patient migration, two third of respondents declared their primary reason was search for quality, the desire to make use of higher quality hospital facilities and medical personnel [5]. These reasons explain the importance to make a Network Campania of Pediatric Oncology (NETCOP) in order to do more efficacy the collaboration between the regional centers of pediatric oncology (Hub centers) and pediatric hospitals present in the five province of Campania region (Spoke centers). In the next future other problem is the recruitment of immigrant children that underwent a progressive, steady increase over the years, growing from 30 cases (2 % of the total number) in 1999 to 130 cases (8 % of the total number) in 2008 [8–10]. Actually, AIEOP centers in Campania treated 2.4 % of immigrant children [5].

## Conclusions

The present findings update descriptive cancer epidemiology in children (0–15 years old) in Campania based on data provided by specialized clinical Mod.1.01 from AIEOP centers and showed an increase over time of O/E ratio, probably due to improvement in the organization of centers and greater trust of families in local centers. Findings derived from this analysis suggest that it is possible to further improve the efficiency of healthcare system of Campania region and migration, problem actually still relevant for the specific cancers that can be reduced with a more rational use of hospitals throughout region.
